# Health Impacts of Ambient Air Pollution in Finland

**DOI:** 10.3390/ijerph15040736

**Published:** 2018-04-12

**Authors:** Heli Lehtomäki, Antti Korhonen, Arja Asikainen, Niko Karvosenoja, Kaarle Kupiainen, Ville-Veikko Paunu, Mikko Savolahti, Mikhail Sofiev, Yuliia Palamarchuk, Ari Karppinen, Jaakko Kukkonen, Otto Hänninen

**Affiliations:** 1 National Institute for Health and Welfare (THL), 70701 Kuopio, Finland; antti.korhonen@thl.fi (A.K.); arja.asikainen@thl.fi (A.A.); otto.hanninen@thl.fi (O.H.); 2 Finnish Environmental Institute (SYKE), 00251 Helsinki, Finland; niko.karvosenoja@ymparisto.fi (N.K.); kaarle.kupiainen@ymparisto.fi (K.K.); ville-veikko.paunu@ymparisto.fi (V.-V.P.); mikko.savolahti@ymparisto.fi (M.S.); 3 Finnish Meteorological Institute (FMI), 00560 Helsinki, Finland; mikhail.sofiev@fmi.fi (M.S.); yuliia.palamarchuk@fmi.fi (Y.P.); ari.karppinen@fmi.fi (A.K.); jaakko.kukkonen@fmi.fi (J.K.)

**Keywords:** disease burden, mortality, morbidity, particulate matter, fine particles, ozone, nitrogen dioxide

## Abstract

Air pollution has been estimated to be one of the leading environmental health risks in Finland. National health impact estimates existing to date have focused on particles (PM) and ozone (O_3_). In this work, we quantify the impacts of particles, ozone, and nitrogen dioxide (NO_2_) in 2015, and analyze the related uncertainties. The exposures were estimated with a high spatial resolution chemical transport model, and adjusted to observed concentrations. We calculated the health impacts according to Word Health Organization (WHO) working group recommendations. According to our results, ambient air pollution caused a burden of 34,800 disability-adjusted life years (DALY). Fine particles were the main contributor (74%) to the disease burden, which is in line with the earlier studies. The attributable burden was dominated by mortality (32,900 years of life lost (YLL); 95%). Impacts differed between population age groups. The burden was clearly higher in the adult population over 30 years (98%), due to the dominant role of mortality impacts. Uncertainties due to the concentration–response functions were larger than those related to exposures.

## 1. Introduction

Air pollution was recognized as a health risk factor by World Health Organization (WHO) already in 1958 [1], but lack of data and related methodologies prevented development of specific recommendations at that time. The first air quality guidelines for Europe were published in 1987 [2] and updated in 2000 [3], when fine particles (PM_2.5_) was given an exposure–response relationship without a threshold. So far, the latest air quality guideline “The global update 2005”, focused on four pollutants PM, O_3_, NO_2_, and sulfur dioxide (SO_2_), and for the first time, set a guideline level for PM_2.5_ [4].

Due to adverse health effects of PM_2.5_, WHO set a guideline to annual mean of 10 µg/m^3^. This guideline is based on evidence of total cardiopulmonary and lung cancer mortality increases, with more than 95% confidence, due to long-term exposure to PM_2.5_ concentrations higher than 10 µg/m^3^. Since then there has been a growing evidence of adverse health impacts also at lower concentrations than 10 µg/m^3^ (e.g., [5,6]). Consequently, the WHO working group, which reviewed the latest scientific evidence for health effects of air pollution [7], recommended updating the WHO guidelines for air quality [8]. 

Quantification of the health impacts helps to compare different risks and to prioritize policy actions. Health impact assessments can also be used to find out the most effective emission mitigation measures to gain the biggest health improvement [9]. In the health impact assessment exposure is combined with population and health data using concentration–response (CR) functions. Ambient air concentrations are often used as proxies for exposure [10]. 

The health risks of air pollution in Europe (HRAPIE) working group [11] gave recommendations for quantifying the adverse health effects of PM_2.5_, respirable particles (PM_10_), O_3_, and NO_2_. In the pollutant–outcomes pairs, there are few newcomers. Chronic mortality was used instead of acute mortality for O_3_. Chronic mortality impacts of NO_2_ were recommended to be calculated for concentrations above 20 µg/m^3^. However, the working group also acknowledged that this level might be too high [12]. 

European Environmental Agency (EEA) has been developing and estimating the exposures and health impacts of ambient air pollution in European counties for over a decade. They use population exposure assessment at 10 km resolution. The assessment combines the European air quality monitoring network data (via AirBase) with spatial interpolation methods, PM_10_/PM_2.5_ modelling to extend the smaller number of PM_2.5_ monitoring sites, and the European Monitoring and Evaluation Programme (EMEP) air quality model at 50 km resolution [13,14,15]. 

EEA’s first health impact estimates were for PM_2.5_ and PM_10_ for 2005 [16]. In the following air quality reports, they started using recommendations of the HRAPIE working group for health impact estimates, and included O_3_ and NO_2_ as well (Table 1). The exposure estimation for NO_2_ was improved for 2014. In the earlier exposure estimates, only background monitoring stations were included, but now also traffic locations were added [17]. This change did not have a large impact on the population-weighted averages, but it affected the exposure distributions. Adding of the traffic stations led to increase in the population at the highest exposure group. Since NO_2_ health impacts were calculated for exposure levels above 20 µg/m^3^, this change had a big impact on the results. 

Early application of air quality models for characterization of health effects was applied for mortality and separating long-range transported particles from the national sources [21,22,23,24]. Long-range transport was estimated by early implementation of the chemical transport model, applied in Finland at 5 km resolution [23]. Unit emission source receptor matrices were developed for simplified estimation of exposures from national sources at 1 km resolution [24]. Mortality related to PM_2.5_ exposure was estimated using relative risks from expert elicitation (1.006–1.01 per 1 µg/m^3^) [25,26], and morbidity outcomes or years of life lost were not estimated. 

Fine particles have been estimated to be the leading environmental risk factor in Finland (e.g., [27,28]). National health impact estimates, accounting also for morbidity, were developed based on methods used in the Clean Air for Europe (CAFE) program [29] in 2010–2011 in [30,31]. The health impacts were discounted and age weighted, giving less weight on health impacts happening far in the future and on very young or old populations. A constant discount rate of 3% was used in the background health data. This means that the health impacts happening in future were valued 3% less each year. In European Perspectives on Environmental Burden of Disease (EBoDE) study, the health impacts were calculated with and without discounting. Removing discounting more than doubled the disease burden attributable to PM_2.5_ (11,000 to 24,000 DALY/a) in Finland [31].

In this work, we estimate the health impacts of ambient air pollution in Finland in 2015. According to the WHO recommendations, we add NO_2_ to the estimated pollutants, and for the first time apply spatially resolved exposure data at 1 km resolution together with burden of disease methodology. Specifically, we (i) estimate the exposures to PM_2.5_, PM_10_, NO_2_, and O_3_; (ii) quantify and compare health impacts by indicator pollutant and by health endpoint; (iii) assess the respective age and gender distributions; (iv) conduct parametric uncertainty and sensitivity analysis for exposure estimation and relative risks; and (v) discuss the sensitivity of NO_2_ estimates to the choice of a cut-off level.

## 2. Material and Methods 

In this work, exposure is defined as the annual average population-weighted ambient air concentration. We calculated the concentrations based on high resolution System for Integrated modelLing of Atmospheric coMposition (SILAM) chemical transport model, as described below. The health impact assessment was done for Finland, for 2015, using disease burden methods and population attributable fractions. 

### 2.1. Emission Estimates and Air Quality Modelling

The exposure estimation for PM_2.5_, PM_10_, NO_2,_ O_3_, and SOMO35 was done using SILAM v5.5 dispersion model [32]. SILAM has previously been extensively evaluated against monitoring data, and demonstrated fairly good agreement with slight underestimation of PM concentrations [33]. This system was developed in Finnish Meteorological Institutes (FMI), and is currently exploited and evaluated on a daily basis as part of the European-scale Copernicus AQ service [34] and in the national operational service, providing the air quality forecasts on global/European/Finnish spatial scales [35]. 

**Figure 1:**
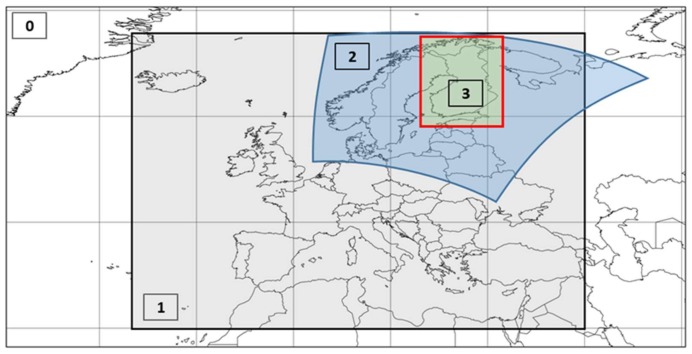
System for Integrated modelLing of Atmospheric coMposition (SILAM) computational domains: 0—global domain, 1—European (resolution 50 km), 2—Northern Europe (Fennoscandia, resolution 10 km), 3—Finland (resolution 1 km).

To compute the concentrations over Finland with horizontal resolution of 1 km, the set of nested SILAM simulations was performed for the whole year 2015. SILAM has four computational domains which are presented in Figure 1. The largest domain was global with 1.44 degrees resolution covering troposphere and the stratosphere (domain 0, model time step 30 min). The calculations over Europe with 50 km grid cell covered only the troposphere (domain 1 with time step 15 min) and were nested into the global run. The Northern European domain, with the resolution of 10 km and time step of 6 min, finally laid down the basis for the main simulations of the study: it generated physically realistic boundary conditions for the model run on the innermost domain over Finland (domain 3). 

The high-resolution computations have the internal model time step of 1.5 min and hourly-mean fields with 0.02° latitudinal and 0.01° longitudinal grid steps. In the vertical direction, the setup included eleven layers of thickness, growing from 20 m near the ground up to 2000 m in the upper troposphere. 

The chemical transformations of atmospheric constituents included extended carbon-bond mechanism CBM4 [36] for gas phase reactions, formation of secondary inorganic aerosols following the updated mechanisms of [37], whereas organic aerosols were handled via the volatility-basis set of multi-phase reactions. Wet and dry depositions from the atmosphere to the underlying surface were calculated on every time step following [38]. 

The meteorological fields, the main drivers of dispersion and chemical transformation, were obtained from the European Centrum of Medium range Weather Forecast (ECMWF [39]) integrated forecast system (IFS) for all regional runs and from ECMWF re-analysis ERA-Interim for the global run. These data had the spatial resolution of about 15/80 km and 3 h update frequency, respectively.

The high resolution modelling relied on the national emissions provided by Finnish Environment Institute (SYKE) from the Finnish Regional Emission Scenario (FRES) model with 250 m spatial resolution for areal sources and an comprehensive list of point emissions (*n* = 581) in Finland [40]. The Ship Traffic Emission Assessment Model (STEAM) [41] supplied the time-resolved ship emission fields (1 km, 1 h) based on tracking of every vessel in Baltic Sea and main harbors and rivers using the anti-collision Automatic Identification System (AIS) reports. To describe the emissions outside of Finland, the updated TNO CAMS (the Netherlands Organisation for Applied Scientific Research, Copernicus Atmospheric Services) inventory [42] was used. The gaseous biomass burning emissions originated from the European vegetation fires were included with the use of Global Fire Assimilation System (GFAS) [43] (data contained modified Copernicus Atmosphere Monitoring Service Information for 2015). The fire-induced particulate matter emission was taken from the FMI IS4FIRES (Integrated Monitoring and Modelling System for wildland fires) database, as described in [44]. 

The PM load from the desert dust was directly computed by SILAM on the global and European domains, and taken into account in the nested regions via transport through the boundaries. Model parameterization of dust emission is based on the modified approaches of [45,46].

Hourly ozone concentrations delivered by SILAM were used to compute the yearly SOMO35 exposure metric as the sum of exceedances over 35 ppb (70 µg/m^3^) of daily maximum 8 h running averages. All the health effects calculated here for ozone are based on SOMO35 levels.

Population counts at 1 km resolution presented in ETRS-TM35FIN coordinate reference system were obtained from Statistics Finland for 31 December 2015 (total population 5.4 million) [47]. The number of total population is available for all inhabited cells (100,338). The cells, which contain at least 10 persons (34,399 cells covering 5.17 million people), include also numbers of men and women, and numbers of people under 15 years old, 15–64 years old, and over 65 years old. 

Air quality monitoring data maintained by municipalities, Helsinki Region Environmental Services Authority (HSY), industry, and FMI were available for 37 PM_2.5_ stations (including 13 background, 15 traffic, and 7 industrial stations), 62 PM_10_ (15/34/13), 64 NO_2_ (21/28/13), and 21 O_3_ (18/2/1) stations located in 50 municipalities [48]. The observed data from the monitoring stations are used for adjustment of the predicted concentrations. 

### 2.2. Exposure Assessment by Adjustment of Predicted Concentrations

SILAM model results (C_pred_) for all modelled pollutants (PM_2.5_, PM_10_, NO_2_, O_3_, and SOMO35) were compared with the observations from the air quality monitoring network (C_obs_). This was done using nearest modelling point. Fractional biases (FB) were calculated to characterize the bias between the observed and predicted concentrations [49]. Spatial processing was conducted with QGIS (2.14.13 Essen, 2017) software [50].

Population-weighted average concentrations (*PWC_pred_*) for the whole country were calculated by using the gridded population as weights for the modelled concentrations. *PWC_pred_* were adjusted for the difference between the observed and predicted concentrations using Equation (1):

(1) $$PWC_{adj}=\;PWC_{pred}+\left(C_{obs}-C_{pred}\right)$$

where *PWC_adj_* is the population-weighted average exposure used in the health impact calculation. Standard deviations (*SD*) of the predicted population-weighted concentrations (*PWSD_pred_*) were adjusted for the obs/pred ratio using Equation (2):

(2) $$PWSD_{adj}=\;PWSD_{pred}\times \frac{SD_{obs}}{SD_{pred}}$$

NO_2_ maximum daily 1 h exposures (NO_2max1ha_) were estimated from the annual average using the daily 1 h max/daily mean ratio observed at the 21 background stations (the ratio was similar also at traffic and industrial monitoring stations).

### 2.3. Quantification of the Health Impacts

The health impacts of air pollution were calculated using a disease burden model called ISTE. It utilizes disease burden methods [9] which are suitable for assessing the health impacts of multiple risk factors at population level. The model contains a background burden of disease modules with currently 26 diseases and groups chosen from the Global Health Estimates (GHE) data and an additional 15 reorganized categories. The model also includes a library of identified relevant concentration–response relationships (currently ca. 200). The library includes the concentration–response functions from the HRAPIE recommendations [12] as well.

WHO Global Health Estimates (GHE) 2000–2015 data [51] were used for the background mortality and morbidity without discounting. Calculations are based on the standard life table, which assumes life expectancy of 92 years at birth, according to projected frontier life expectancy for 2050. Data for Finland were extracted from the database containing estimates for 183 countries.

The attributable disease burden (AB) was calculated combining population attributable fraction (PAF) with background disease burden (BoD) (Equation (3)).

(3) AB=PAF×BoD

where PAF is calculated as

(4) $$\mathrm{PAF}=\frac{f\times \left(RR_{E}-1\right)}{f\times \left(RR_{E}-1\right)+1}$$

in which f is the percentage of the exposed population in the whole target population. *RR_E_* is the relative risk of the population at the prevailing exposure level, calculated as

(5) $$RR_{E}=RR_{1}^{E}$$

in which *RR*_1_ is the relative risk estimate per unit of exposure and *E* is the exposure in the population level. *PWC_adj_* values were used as exposures. 

Relative risks were selected based on the WHO HRAPIE recommendations [12]. In the recommendations, concentration–response functions were classified to A and B classes regarding how reliable the function is, A being more reliable than B. The functions which are possible to sum up together were marked with an asterisk (*). The functions we chose for the health impact calculations are presented in Table 2. We calculated natural mortality as all-cause mortality, excluding injuries and violent causes of death. NO_2_ health effects were assumed to be 33%, overlapping with PM_2.5_ health effects [12], and therefore, they only accounted for 67%. The functions were assumed to be log-linear. 

Hospital admissions were quantified using the GHE data like for other endpoints as well. Morbidity (YLD) of cardiovascular diseases (GHE code 110) and respiratory diseases (118) were used for baseline health data instead of hospital admission data. 

The results are mainly expressed as disability-adjusted life years (DALY) and number of deaths. Also, the ratio between years of life lost (YLL) due to premature mortality and years lived with disability (YLD) are presented. Use of common units enables comparisons between risk factors and different health outcomes. 

### 2.4. Uncertainties and Sensitivity Analysis

The uncertainties related to exposure were determined by first calculating the standard errors (SE) at the available monitoring sites and coefficients of uncertainty (CU) for each pollutant:

(6) $$\mathrm{SE}=\frac{{\mathrm{SD}}_{\mathrm{obs}}}{\sqrt{n}}$$

(7) $$\mathrm{CU}=\frac{\mathrm{SE}}{C_{\mathrm{obs}}}$$

where SD_obs_ is standard deviation of the monitoring data and n is the number of monitoring stations. C_obs_ is the annual mean observed concentration. CU is then applied to the adjusted concentrations to calculate confidence intervals (CI) for the population exposures:

(8) $$\mathrm{CI}\left(\pm 95\text{\%}\right)=\left(1\pm 1.96\mathrm{CU}\right)\times {\mathrm{PWC}}_{\mathrm{adj}}$$

Parametric uncertainty was expressed using 95% confidence intervals for the health impact estimates, calculated by combining the uncertainties in both relative risk (Table 2) and exposure parameters (PWC 95% CI, presented in the Section 3.1).

For a sensitivity analysis, the probability density distribution of exposures were also generated by adjusting the standard deviation of exposures for corresponding observed/predicted ratios at the available monitoring sites for each pollutant. Specifically, for quantifying the sensitivity of NO_2_ effects on the selected cut-off values, the whole estimated population exposure distribution was first stretched according to the underprediction of standard deviation at the monitoring sites. The spread adjustment factor, *k*, was calculated from the NO_2_ monitoring locations as

(9) $$k=\frac{SD_{obs}}{SD_{pred}}$$

and applied to concentrations *C_i_* at the occupied grid cells according to

(10) $$C_{i,adj.}=C_{adj}+\left(C_{i}-C_{adj}\right)\times k$$

where *C_adj_* is the mean population weighted and adjusted NO_2_ concentration.

Sensitivity of the natural mortality impacts related to NO_2_ exposure was estimated using selected cut-off levels (10, 15, and 20 µg/m^3^). The mortality impacts were calculated using the exposure data in the inhabited (100,338) grid cells from SILAM model adjusted for fractional bias. The years of life lost and number of premature deaths were estimated for each cell, as described earlier, and summed up to get the total impacts in the whole country. 

## 3. Results

Population-weighted concentrations using SILAM model with adjustment to the observed concentrations were used to estimate health effects with disease burden methods. Results are presented by indicator pollutant, health outcomes, and population age groups. 

### 3.1. Exposure Estimates of Ambient Air Pollution in 2015

The modelled annual concentrations were, on average, slightly lower than the observed ones when compared at the locations of the measurement stations (Table 3). PM_2.5_ concentrations had the lowest fractional bias, while bias was higher for PM_10_, NO_2_, and O_3_, and considerably higher for SOMO35.

The adjusted population-weighted concentration for PM_2.5_ was only slightly higher when compared to unadjusted while for the other components the adjustment plays a substantial role and seems essential (Table 4). 

### 3.2. Attributable Disease Burden

**Figure 2:**
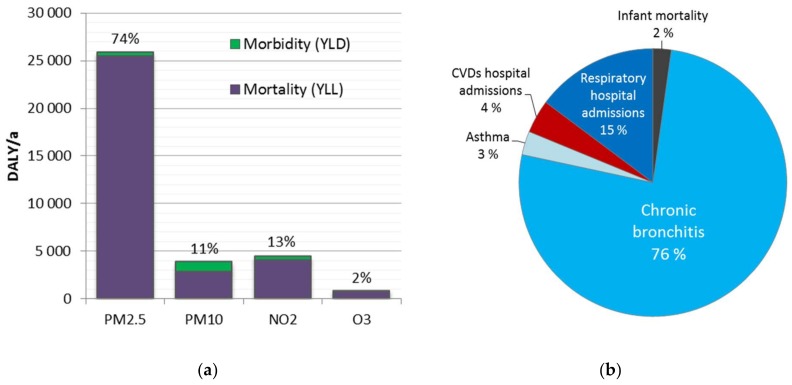
(**a**) Attributable disease burden for the four assessed air pollutants in Finland (35,000 DALY in 2015). (**b**) Disease burden by health outcomes excluding natural mortality (4600 DALY).

The disease burden attributable to the four pollutants was 35,000 DALY in 2015 (Table 5). That is 2% of the total disease burden in Finland (1,530,000 DALY). Air pollution was associated with 2000 premature deaths (1400–2600 95% CI). The largest part (74%) of the disease burden was associated with PM_2.5_ exposure. Mortality was dominant over morbidity effects for all pollutants (Figure 2a).

The disease burden of the considered air pollutants is dominated by mortality rather than morbidity (95%). This is due to the fact that the largest part of the disease burden is caused by natural mortality. The morbidity component is strongly driven by respiratory diseases, especially chronic bronchitis (Figure 2b). Hospital admissions due to respiratory diseases also had a notable share of the disease burden. Hospital admission due to cardiovascular diseases, asthma, and infant mortality had the smallest share of the total attributable disease burden.

**Figure 3:**
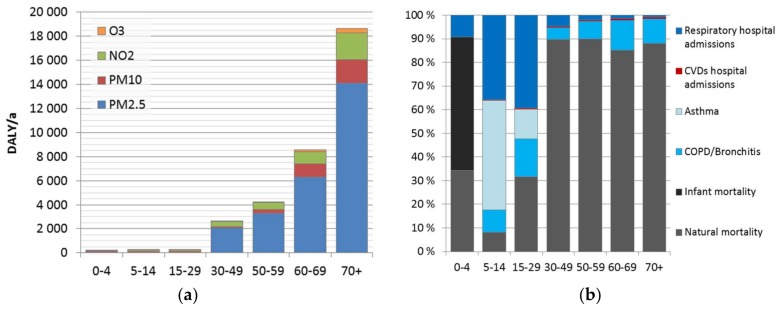
(**a**) Disease burden of three air pollutants in seven age groups in Finland in 2015. (**b**) Health outcomes caused by ambient air pollution in age groups.

Health impacts of air pollution increase strongly above 30 years of age (Figure 3a). After infancy, at younger age groups (5–30 years) mortality is rare, while for other age groups (<5 years and >30) it is the dominant endpoint (Figure 3b).

Finnish gender distribution is nearly even (51% female), but the disease burden is higher in males (53% of total; 66% of lung cancer; 58% cardiovascular disease; and 55% respiratory diseases). Hence, the disease burden attributable to air pollution is also weighted more on men (57%).

### 3.3. Uncertainties and Sensitivity Analyses

**Figure 4:**
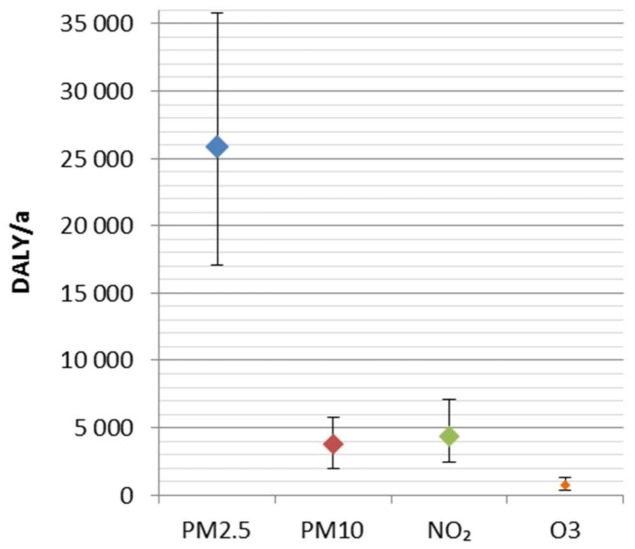
Combined uncertainties from exposure and relative risks for disease burden of ambient air pollution as disability-adjusted life years (DALY) in 2015 in Finland.

The uncertainties originating from the relative risk estimates were larger than the ones related to exposure assessment. This is also in line with an earlier study [52]. Confidence intervals (95%) due to exposure were 32,600 to 37,000 DALY/a, and due to exposure–response relationship, 25,400 to 43,400 DALY/a for the attributable disease burden of air pollution. When both of these uncertainties were accounted for, the combined 95% confidence intervals were 25,400 to 45,500 DALY/a. The combined uncertainty intervals per pollutant are presented in Figure 4. The relative combined uncertainties for PM_2.5_, PM_10_, NO_2_, and O_3_ were −34–39%, −49–51%, −44–62%, and −59–79%, respectively. The relative uncertainties were smallest for PM_2.5_ and largest for O_3_.

We also compared the disease burden attributable to air pollution estimated using a point value for exposure with estimation where exposure distribution is taken into account. Accounting for the probability distribution of exposures led to 3.8% higher results. 

## 4. Discussion

In the recent Global Burden of Disease study, air pollution was evaluated as one of the most important environmental health risk factors [53]. In our work, we focused on the health impacts of four ambient air pollutants. We estimated the exposure to air pollution in Finland in 2015 using a state of art chemical transport model with high spatial resolution. The predicted concentrations were further adjusted for the difference of the observed and predicted concentrations at nationally available monitoring stations. The impacts of adjustment were small (ca. +5%) for PM_2.5_ concentrations, but for the other components (NO_2_, PM_10_, SOMO35) much more substantial (Table 3), and therefore, here, deemed essential. 

We estimated 1,500 deaths were related to PM_2.5_ exposure in 2015. This is of the same magnitude with the number of deaths attributable to PM_2.5_ estimated by EEA (Table 1). The number of deaths estimated by EEA, available for the period 2005–2014, range from 1700 to 2500, highlighting the variability of exposures between consecutive years. The current and ISTE estimates for years of life lost are based on the WHO Global Frontier 2050 projections for life expectancy, which is roughly 10 years higher than the current national one. This leads to substantial difference in comparison with YLL figures of EEA.

In comparison to earlier national estimates for PM_2.5_, the differences reflect (i) temporal trends in exposures from 2000 to 2015; (ii) differences and developments in exposure estimation methods (including chemical transport modelling, source receptor matrices, and use of monitoring network data); and (iii) differences in the concentration–response modelling (Table 6). 

EEA has recognized the problematic nature of cut-off values and has calculated NO_2_ estimates with cut-off values of 10 and 20 µg/m^3^, showing that the lowering of the cut-off value had an 11-fold increase of the number of estimated deaths in Finland [20]. Problems arise e.g., when averaging exposures between areas that are partly below and partly over the cut-off level, leading to a “no effects” estimate.

We evaluated NO_2_ mortality using concentration–response function without a threshold (RR = 1.0027 (Table 2)). WHO recommendations [12], though, included also a significantly higher relative risk (1.055) for NO_2_ related mortality, based on a cut-off level of 20 µg/m^3^. This level was given due to larger uncertainties in the relation below 20 µg/m^3^. However, in the later recommendations, they state that this might be too conservative an approach, since there are studies which show a relationship between mortality and NO_2_ exposure at lower levels as well [12]. 

We tested the sensitivity of the NO_2_ mortality (RR = 1.055) using three cut-off levels (Table 7). We found that the differences between shown estimates are huge (e.g., 23 vs. 1200 deaths; 370 vs. 20,000 YLL), concretely showing that this scientifically quite uncertain response model parameter has an utmost importance for the numerical results.

The fractional bias of SOMO35 was expectedly large. This is a result of the low stability of all threshold-based indexes, which was pointed out by [54]. It explains the disproportional reaction of the index to the SILAM underestimation of ozone, of which traces remain, even after the simple bias correction. Secondly, our SOMO35 index is lower than that computed by EEA because the latter is based on monitoring data, all but three are located in background locations, and thus, show higher levels than those prevailing in the urban populated areas. It is actually likely that the monitoring station-based EEA exposure estimates may be too high. Here, the chemical transport model, in principle, accounts for the degradation of ozone in urban areas, and may, thus, contain information missing from the EEA model.

In our calculations, we used observed concentrations from all monitoring stations (*n* = 21) as in previous estimates (i.e., [55]). The majority of the stations are background stations which have higher ozone levels than urban ones, leading to a risk of overestimation. We did sensitivity analysis for the SOMO35 concentrations in different station types. The population lives mainly in urban and suburban areas, so we calculated the PWC using stations located in those areas (*n* = 7). We also calculated the concentration on rural background stations (*n* = 11) to find out how much it differs from the urban/suburban stations. The adjusted population-weighted concentrations for urban/suburban areas and for rural background stations were 740 µg/m^3^ and 1400 µg/m^3^, respectively, while the PWC for all stations was 1040 µg/m^3^. Concentrations from the all stations might overestimate the exposure up to 41%.

The SILAM model is known to underestimate O_3_ concentrations during warm seasons, and therefore, the seasonal peculiarities need to be further examined. Here, the adjustment to observed concentrations should, though, correct this underestimation.

Recent updates in the disease burden methodology, especially dropping off discounting and age weighting by the global assessment community, as well as projected changes in life expectancy, have increased the disease burden estimates. This increases the estimates in comparison to those evaluated earlier using WHO GBD 2004 disease burden data. Current work uses WHO Global Frontier 2050 life expectancies (92 years at birth), which is substantially larger than national life expectancy in Finland (84 years for females and 79 years for males in 2015) [56]. 

In the current approach, relative risks are assumed to be same for male and female [12]. We also assumed same exposure over different age groups and genders. This is a clear weakness of our analysis. However, we did some primary analysis related to differences in PM_2.5_ exposure between age groups and genders, and found differences less than 2% of the mean PM_2.5_ exposure on the population level. Although, it is possible that regionally larger differences exist. We will investigate these in more detail in a geospatial follow-up study, while in the current work, the same exposures were used for both genders and ages. 

We found that the minor differences in the attributable burden between the genders are caused by the background disease burden. Much larger differences were found between population age groups, caused by age-dependence of the background disease burden, and included pollutant–outcome pairs. Overall, currently estimated impacts of air pollution increase heavily on population over 30 years, representing 98% of the total attributable burden. This is mainly due to mortality impacts. Use of natural mortality as an endpoint does not include the corresponding morbidity. The evaluation is lacking other effects, such as learning/academic performance of children and students, or productivity at work. These effects should be elaborated in more detail, to form a more comprehensive picture of the overall effects of air pollution.

The use of point value for exposure leads to numerical differences in the results when using non-linear exposure–response relationships, such as the traditional log-linear function (e.g., [27]) or the integrated exposure response (IER) function (e.g., [57]). The non-linear IER functions have been used in the recent global disease burden assessments of air pollution [57,58]. As part of the sensitivity analyses, we quantified these using an assumed normal distribution with variance estimated from the adjusted SILAM data, showing that in the current work, the use of point value leads to an underestimation by 3.8% when log-linear functions are used. Difference can be higher, though, in cases where a cut-off is used, and the exposure is close to the cut-off level. The IER functions include a cut-off level. Use of IER functions without taking into account the exposure distribution in areas where the concentrations are low can lead to substantially lower health impact estimates than when log-linear functions are used. 

Another commonly used approach to handle exposure variability is related to spatial modelling (e.g., [57,59]), where population is allocated to grid cells and respective exposure levels. Such an approach may underestimate exposure variance due to deterministic point value modelling approaches, or due to coarse spatial resolution. Comparison of the benefits from spatial variation versus model error and embedded underestimation of variance needs to be further elaborated, but tentative interpretation of the current results suggest that the probability distribution approach works well.

Ambient air contains a mixture of contaminants, and therefore, we are exposed to different air pollutants at the same time. PM, NO_2_, and O_3_ correlate with each other to some extent, and their health impacts can overlap. WHO recommended different health endpoints for PM_2.5_ and PM_10_, which helps to avoid double-counting. Some of the NO_2_ long term effects were thought to overlap with particles up to 33%, which was taken into account in our analysis.

Air pollution has been estimated as the leading environmental risk factor in Finland, and particles as the most important air pollutant [27,60]. The latter is well confirmed in the current results. Especially the use of PM_2.5_ and PM_10_ exposures in the assessment highlights their indicator role—it cannot be interpreted that PM_10_ effects would be the sum of fine and coarse particles. PM_10_ is merely used as an alternative—often less reliable, but more widely available—exposure indicator.

## 5. Conclusions

In this work, concentrations of PM_2.5_, PM_10_, O_3_, and NO_2_ were estimated for Finland for 2015 using chemical transport model (SILAM) with adjustment to monitoring data. The concentrations were population weighted at 1 km resolution and used as exposure estimates. Resulting exposure levels were slightly smaller than averages of the air quality monitoring stations (e.g., −0.5 µg/m^3^ for PM_2.5_). 

The disease burden attributable to the four ambient air pollutants was 34,800 DALY (25,400–45,500 95% CI). It represents ca. 2% of the total national burden of disease, and includes 2000 (1400–2600 95% CI) premature deaths. A major share (74%) of the disease burden was attributed to fine particles (PM_2.5_). The results are consistent with the previous comparative risk assessments, which have suggested fine particles as the leading environmental risk factor in Finland. 

Disease burden was significantly higher at adult age groups, especially after 30 years old (98%). This is strongly related to the dominant role of mortality, which increases as a function of age. Before 30 years old, morbidity was 60% of the disease burden, while for age groups above 30 years, it was only 4.5%. However, morbidity impacts might be underestimated. It may be useful to improve the estimation and investigate also impacts on learning, academic performance, and productivity.

The uncertainties related to concentration–response functions were larger than ones related to the exposure. Overall, in the ranking of particles, NO_2_ and O_3_ seems robust, regardless of the uncertainties. In order to improve the estimation of health impacts, the shape of concentration–response functions, especially at current low exposure levels, should be studied in more detail, e.g., using health register-based epidemiological methods.

## Figures and Tables

**Table 1:** European Environmental Agency (EEA) estimates for premature deaths related to ambient air pollution exposure in Finland.

Pollutant	Annual Estimated Premature Deaths			
	2005	2012	2013	2014
PM_2.5_	2500	1900	1700	2200
O_3_	n/a	60	80	60
NO_2_	n/a	0	<5	40
References	[16]	[18]	[19]	[20]

n/a not assessed.

**Table 2:** Relative risk (RR) estimates with confidence intervals used in this work [12].

Pollutant	Health Endpoint	Ages	RR per 10 µg/m^3^ (95% CI)	a
PM_2.5_	Natural mortality	>30 year	1.062 (1.040–1.083)	A*
	CVDs (hospital admissions)	all	1.0091 (1.0017–1.0166)	A*
	Respiratory (hospital admissions)	all	1.0190 (0.9982–1.0402)	A*
PM_10_	Infant mortality	1–12 month	1.04 (1.02–1.07)	B*
	Chronic bronchitis (children)	6–12 year	1.08 (0.98–1.19)	B*
	Chronic bronchitis (adults)	>18 year	1.117 (1.040–1.189)	B*
	Asthma symptoms (children)	5–19 year	1.028 (1.006–1.051)	B*
NO_2_	Natural mortality ^b^	all	1.0027 (1.0016–1.0038)	A*
	Natural mortality (>20 µg/m^3^) ^c^	>30 year	1.055 (1.031–1.080)	B*
	Bronchitis symptoms	all	1.021 (0.990–1.060)	B*
	Respiratory (hospital admission)	all	1.0180 (1.0115–1.0245)	A*
O_3_	Natural mortality	all	1.0029 (1.0014–1.0043)	A*
	CVDs (hospital admissions)	>65 year	1.0089 (1.0050–1.0127)	A*
	Respiratory (hospital admissions)	>65 year	1.0044 (1.0007–1.0083)	A*

^a^ Additivity category, see text and [12] for definition. ^b^ Calculated for NO_2_ max 1 h annual averages. ^c^ Used for sensitivity analysis for NO_2_ mortality impacts.

**Table 3:** Mean observed (obs) and predicted (pred) concentrations at monitoring stations and fractional bias (FB) of unadjusted predictions.

Pollutant	Stations *n*	Obs (±SD) µg/m^3^	Pred (±SD) µg/m^3^	FB %
PM_2.5_	37	5.8 (±1.4)	5.6 (±1.3)	−5
PM_10_	62	13 (±4.3)	6.7 (±1.9)	−60
O_3_	21	53 (±7.5)	41 (±4.5)	−25
SOMO35	21	1200 (±590)	220 (±150)	−140
NO_2_	64	13 (±8.3)	8.8 (±4.3)	−39
NO_2max1ha_	64	28 (±15)	–	–

SD = standard deviation, FB = fractional bias.

**Table 4:** Adjusted population-weighted concentrations (PWC_adj_) in Finland in 2015.

Pollutant	PWC_pred_ µg/m^3^	PWC_adj_ (95% CI) µg/m^3^		SE µg/m^3^	SD µg/m^3^
PM_2.5_	5.1	5.3	(4.9–5.8)	0.22	1.4
PM_10_	6.2	12	(11–13)	0.52	4.2
SOMO35	99	1040	(810–1300)	120	170
NO_2_	7.8	12	(10–14)	0.96	8.9
NO_2_1hmax_	–	26	(22–31)	2.3	21

PWC = Population-weighted concentration; CI = confidence interval; SE = standard error, SD = standard deviation.

**Table 5:** Estimates of attributable disease burden for four main air pollutants in Finland in 2015.

Pollutant	DALY	(95% CI)	YLL/YLD	Deaths	YLL/Death
PM_2.5_	26,000	(17,000–36,000)	61	1600	16
PM_10_	3800	(1900–5700)	3	160	18
NO_2_	4400	(2400–7100)	10	240	17
O_3_^a^	750	(330–1300)	11	40	17
Total	35,000	(25,000–46,000)	17	2000	17

DALY disability-adjusted life years, YLL years of life lost, YLD years lived with disability, CI confidence interval. ^a^ Ozone impacts are based on SOMO35.

**Table 6:** National studies which have evaluated the health impacts of PM_2.5_ in Finland.

Project	KOPRA ^a^	PILTTI ^a,b^	EBoDE	SETURI ^c^	ISTE	BATMAN
Year ^d^	2000	2000	2005	2005	2013	2015
Exposure resolution	SILAM 5 km	FRES 1 km	EEA 10 km	EEA 10 km	Monitoring	SILAM 5.5 1 km
Exposure (µg/m^3^)	0.8 ^a^	2.5 ^a,b^	9.1	9.1	6.8	5.3
Deaths (n)	350	1000	1700 ^e^	1800	980 ^f^	1500
Burden (DALY)	n/a	n/a	24,000	14,000 ^c^	18,000	26,000
References	[23]	[24]	[27,31]	[28]	[54,55]	Present paper

n/a not assessed in the study; ^a^ only primary particles; ^b^ only national sources; ^c^ discounted values; ^d^ target year of the assessment; ^e^ not reported in the source, figure obtained by communication with the authors; ^f^ includes only cardiopulmonary deaths and lung cancer.

**Table 7:** Mortality impacts of nitrogen dioxide at different cut-off levels and RR = 1.055.

Cut-off µg/m^3^	Years of Life Lost YLL	Deaths *n*
10	20,000	1200
15	11,000	650
20 ^a^	370	23

^a^ Working group recommendation [12].
